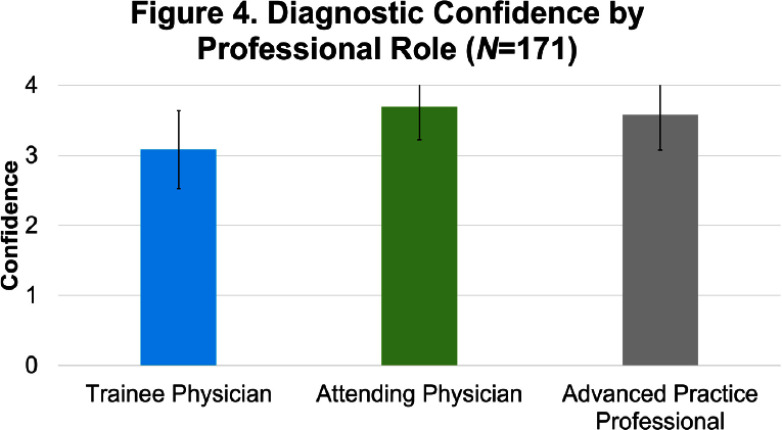# Lower Clinician Confidence is Associated with Longer Intended Treatment Duration for Outpatient Men with Urinary Tract Infections

**DOI:** 10.1017/ash.2025.247

**Published:** 2025-09-24

**Authors:** Tyler Brehm, Larissa Grigoryan, Laura Dillon, Trenton Haltom, Barbara Trautner

**Affiliations:** 1 Baylor College of Medicine; 2 Baylor College of Medicine; 3 MEDVAMC; 4 Baylor College of Medicine; 5 Baylor College of Medicine

## Abstract

**Background:** There is limited evidence to guide the diagnosis and treatment of urinary tract infections (UTIs) in men. We hypothesized that lower clinician confidence in ability to correctly diagnose or treat UTIs in men would be associated with increased antibiotic treatment duration. **Methods:** We surveyed clinicians’ knowledge and confidence in diagnosing and treating UTIs in outpatient men as well as their intention to prescribe a specific duration of antibiotics. We distributed the survey to outpatient primary care and emergency medicine providers, urologists, and internal medicine residents. We collected demographics on professional role (physician-attending, physician-trainee, advanced practice professionals [APPs]), specialty, and years in practice. Surveys were distributed on paper and electronically. Analysis involved t-test and ANOVA for continuous variables and Chi-squared for categorical variables as appropriate. Multiple logistic regression analyses were performed using the outcome variable of antibiotic treatment duration, categorized as appropriate (5-7 days) or inappropriate (> 7 days). **Results:** 186 of 363 distributed surveys were completed (51% response rate). Fifteen surveys were excluded due to the respondent specialty (e.g., dermatology, neurology, etc.), leaving 171 surveys for analysis. Of these, 60% were from trainees, 26% attendings, and 14% APPs (Figure 1). Most physicians (Figure 2) were internal medicine trained (81%), with a smaller proportion of family medicine (8%), urology (6%), and emergency medicine (5%). 14% of respondents reported an intention to treat UTIs in men for longer than 7 days (Figure 3). Lower clinician confidence in ability to correctly diagnose male UTI was associated with longer intended antibiotic treatment durations (Odds Ratio [OR] 0.42, Confidence Interval [CI] 0.19-0.91, p = 0.03). This association was independent of professional role, specialty, and years in practice. Lower clinician confidence in ability to correctly treat male UTI was not significantly associated with longer intended treatment durations (OR 0.46, CI 0.21-1.03, p = 0.06) on univariate analysis but was significantly associated when adjusted for years since graduation (OR 0.40, CI 0.17-0.96, p = 0.04). Confidence in diagnosis (Figure 4) was significantly different between professional roles, with trainees significantly less confident (median ± standard deviation = 3.1 ± 0.56) than attendings (3.7 ± 0.47, p) **Conclusions:** Lower confidence among clinicians in either diagnosis or treatment of UTIs in men was associated with intention to prescribe longer antibiotic courses. Future studies that address the evidence gaps in diagnosis and management of UTI in men may improve clinician confidence and thus reduce unnecessarily long durations of antibiotics.

**Figure:**
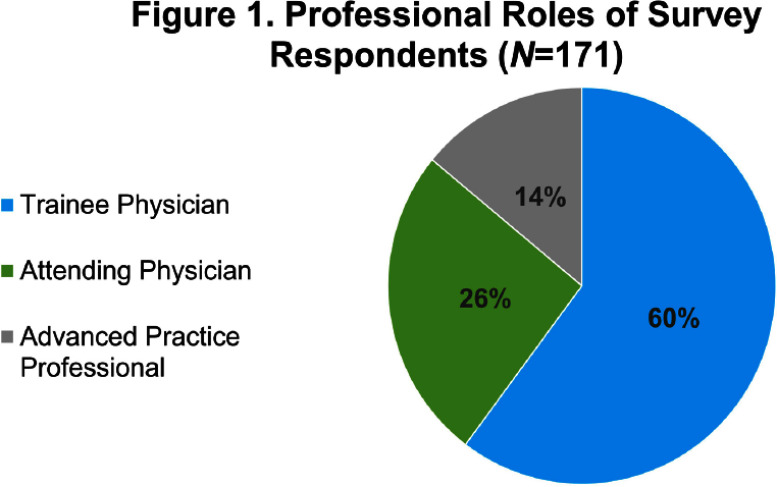

**Figure:**
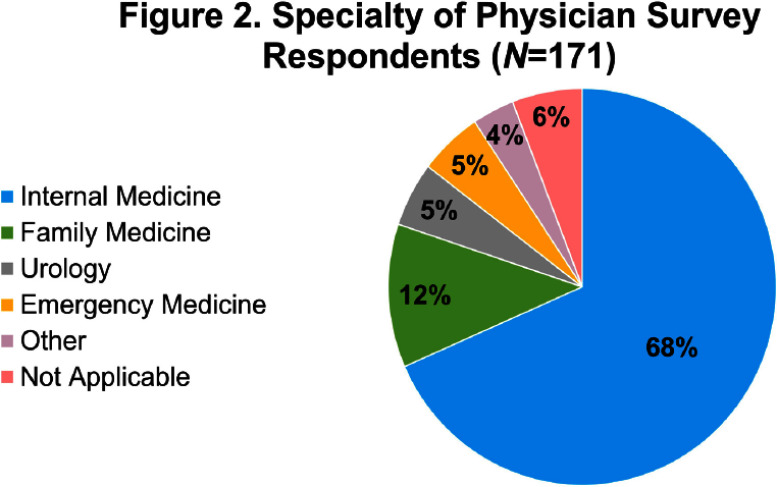

**Figure:**
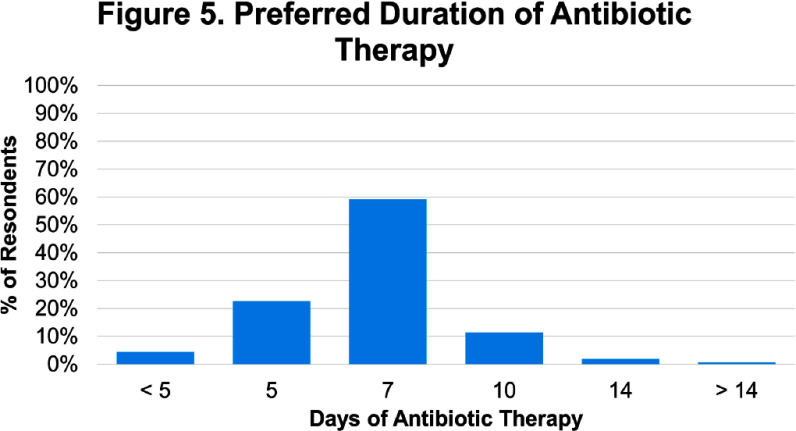

**Figure:**